# The impact of hypotestosteronemia on cardiometabolic effects of atorvastatin in men with hypercholesterolemia: a pilot study

**DOI:** 10.1097/MCA.0000000000001031

**Published:** 2021-03-16

**Authors:** Robert Krysiak, Karolina Kowalcze, Bogusław Okopień

**Affiliations:** a Department of Internal Medicine and Clinical Pharmacology; b Department of Pediatrics in Bytom, School of Health Sciences in Katowice, Medical University of Silesia, Katowice, Poland

**Keywords:** cardiometabolic risk, insulin sensitivity, pleiotropic effects, statins, testicular dysfunction

## Abstract

**Background:**

Hypothyroidism, hyperprolactinemia, macroprolactinemia and low vitamin D status were found to impair pleiotropic effects of hypolipidemic agents. The aim of the current study was to investigate whether cardiometabolic effects of atorvastatin in men are determined by endogenous testosterone.

**Methods:**

We studied three groups of men matched for age, BMI, plasma lipids and blood pressure: 19 untreated subjects with low testosterone levels (group A), 19 normotestosteronemic men receiving testosterone preparations (group B) and 21 untreated men with testosterone levels within the reference range (group C). Because of coexistent hypercholesterolemia, all subjects were managed with atorvastatin (40 mg daily) for 6 months. Glucose homeostasis markers, plasma lipids, as well as circulating levels of testosterone, uric acid, high-sensitivity C-reactive protein (hsCRP), fibrinogen, homocysteine and 25-hydroxyvitamin D were determined at the beginning and at the end of the study.

**Results:**

At baseline, group A was more insulin-resistant and was characterized by higher levels of hsCRP, fibrinogen and homocysteine, and lower levels of 25-hydroxyvitamin D than the remaining groups of patients. Despite reducing total and low-density lipoprotein cholesterol and hsCRP levels in all treatment groups, this effect was stronger in groups B and C than in group A. In groups B and C, atorvastatin use was also associated with a decrease in uric acid, fibrinogen and homocysteine concentrations and with an increase in 25-hydroxyvitamin D levels. In group A, but not in the remaining groups, the drug decreased insulin sensitivity.

**Conclusion:**

The obtained results suggest that untreated hypotestosteronemia may attenuate cardiometabolic effects of atorvastatin in men.

## Introduction

Cardiovascular benefits of 3-hydroxy-3-methylglutaryl-CoA (HMG-CoA) reductase inhibitors (statins) cannot be explained exclusively by lowering lipid levels but also by lipid-independent ‘pleiotropic’ effects, such as antioxidant, antithrombotic, anti-inflammatory, immunosuppressive and endothelial-protective effects, as well as the impact on smooth muscle cell proliferation and migration [1–4]. Earlier studies revealed that pleiotropic effects of statins require normal hormonal status and are less pronounced in subjects in whom dyslipidemia coexists with hypothyroidism [5], monomeric hyperprolactinemia [6], macroprolactinemia [7] or low vitamin D status [8]. Interestingly, the unfavorable effect on statin action was observed in patients with relatively mild stages of endocrine dysfunction. Further impairment of statin action correlated with the degree of each hormonal dysfunction, as well as was reversed if thyroid hypofunction, hyperprolactinemia or vitamin D insufficiency were effectively treated [5,6,8].

Another state which may determine statin action is dysfunction of the hypothalamic-pituitary-testicular axis, a common but often undiagnosed condition in men in developed countries [9]. Our previous study [10], as well as a meta-analysis of randomized controlled trials carried out by other researchers [11] revealed that HMG-CoA reductase inhibitors reduce circulating testosterone levels. This effect, which is probably irrelevant in subjects with undisturbed testicular function, may have clinical significance in hypogonadal men because low circulating testosterone levels are associated with increased cardiovascular mortality and morbidity [12,13]. The unfavorable effect of HMG-CoA reductase inhibitors on testosterone production may explain why intramuscular testosterone enanthane administered to patients with late-onset hypogonadism already treated with atorvastatin, produced multidirectional beneficial cardiometabolic effects, which were much stronger than in atorvastatin-naive men [14]. The aim of the current study was to investigate whether cardiometabolic effects of atorvastatin are modulated by testosterone status.

## Methods

The study was approved by the institutional review board prior to initiation, as well as conducted in accordance with the Declaration of Helsinki. Written informed consent was obtained from all participants after giving them a complete description of the study.

### Study population

The participants of this case–control study (*n* = 59) were recruited among men (40–75 years old) with isolated hypercholesterolemia, defined as total cholesterol levels above 200 mg/dL and low-density lipoprotein (LDL)-cholesterol levels more than 130 mg/dL despite complying with the lifestyle modification for at least three months before entering the study. During these three or more months, all participants had been supervised by local healthcare providers cooperating with our research group. The selection procedure, based on a computer algorithm, was aimed at obtaining three groups of subjects matched for age, BMI, plasma lipids and blood pressure: (1) untreated men with low testosterone levels (*n* = 19); (2) normotestosteronemic men receiving intramuscular testosterone enanthane preparations for at least 6 months because of testicular failure (*n* = 19); (3) untreated men with circulating testosterone levels within the reference range (*n* = 21).^a^ Low testosterone levels were defined as plasma testosterone levels below 10.4 nmol/L, while normal testosterone levels were diagnosed if testosterone concentrations were in the range between 13.9 and 41.6 nmol/L. In order to minimize the impact of seasonal fluctuations in the studied parameters, 30 patients were recruited between December or January and February, whereas the remaining 29 ones were enrolled in June or July.

The exclusion criteria included: testosterone levels between 10.4 and 13.9 nmol/L and above 41.6 nmol/L, diabetes mellitus, thyroid or adrenal disorders, impaired renal or hepatic function, prostate cancer, lower urinary tract symptoms [the American Urological Association (International Prostate Symptom Score exceeding 19)], prostate-specific antigen >4 ng/mL (or >3 ng/mL in men at high risk of prostate cancer), untreated obstructive sleep apnea, hematocrit exceeding 50%, severe myocardial infarction or stroke within 6 months preceding the study, moderate or severe arterial hypertension (European Society of Cardiology/European Society of hypertension grade 2 or 3), heart failure (classes II–IV according to the New York Heart Association Functional Classification), any treatment (except for testosterone) and poor patient compliance. Subjects receiving drugs known to affect plasma lipids or to interact with statins and testosterone were also excluded.

### Study design

All study groups were treated with atorvastatin, administered at the dose of 40 mg once daily at bedtime for 6 months. Throughout the entire study period, the participants continued treatment with the same testosterone preparation, as well as continued to comply with dietary recommendations (<30% of total energy intake, saturated fat intake <7% of energy consumed, cholesterol intake <200 mg per day, an increase in fiber intake to 15 g per 1000 kcal, moderate to vigorous exercise for at least 30 min per day). Over the entire study period, the participants were also recommended to do at least 150 min of moderate-intensity aerobic physical activity per week. Moderate-intensity activity was defined based on the rate of energy expenditure during the activity (between 3 and 6 metabolic equivalents) and on a person’s level of effort (5 or 6 on a scale of 0–10, where sitting is 0 and the highest level of effort is 10). The recommended activities included brisk walking (at least 4 km/h), water aerobics, slow dancing (ballroom or social), tennis (doubles), biking slower than 16 km/h, hiking, washing windows, sweeping the floor, vacuuming or pushing a lawnmower. The participants were also asked to do muscle-strengthening activities that were moderate intensity and involved all major muscle groups on two or more days a week.

Treatment compliance was investigated every 6 weeks by interrogation and pill count. Medication adherence was regarded as satisfactory if the number of tablets returned ranged from 0 to 10% and all four questions in the Polish version of the Morisky-Green test were answered with a ‘no’. Compliance with dietary recommendations and the exercise program was assessed during the same visits, by analysis of individual dietary questionnaires and diaries in which participants continuously recorded all their activities. Moreover, 24-h dietary recall was completed through face-to-face interviews to confirm dietary records.

### Laboratory assays

Venous blood samples were collected from the antecubital vein between 7.00 and 8.00 a.m. after overnight 12-h fasting and assessed in duplicate by a person unaware of the nature of the experiment. Plasma lipids [total cholesterol, LDL-cholesterol, high-density lipoprotein (HDL)-cholesterol and triglycerides], fasting glucose and plasma uric acid were assayed by routine laboratory techniques (Roche Diagnostics, Basel, Switzerland). Plasma levels of insulin, testosterone and homocysteine were measured by direct chemiluminescence using acridinium ester technology (ADVIA Centaur XP Immunoassay System, Siemens Healthcare Diagnostics, Munich, Germany). High-sensitivity C-reactive protein (hsCRP) levels were measured by immunoassay with chemiluminescent detection (Immulite 2000XPi, Siemens Healthcare, Warsaw, Poland). Plasma levels of fibrinogen were assayed by the Clauss technique, using an automated BCS XP analyzer (Siemens Healthcare). Plasma levels of 25-hydroxyvitamin D were detected by competitive immunoassay using a multichannel automatic analyzer (Roche Cobas e 411, Mannheim, Germany). The homeostatic model assessment 1 of insulin resistance (HOMA1-IR) was calculated according to the following formula: plasma glucose (mg/dL) × plasma insulin (mIU/L)/405.

### Statistical analysis

To obtain a Gaussian-shaped distribution, all variables were natural log-transformed. Comparisons between mean absolute values in the study groups, as well as between percent changes from baseline after adjustment for baseline values (reflecting the strength of atorvastatin action) were performed using one-way analysis of variance followed by the post hoc Bonferroni test. Within-group comparisons were made using Student’s paired *t*-tests. Differences in categorical variables were tested using *χ*^2^ tests. Correlations were calculated using Kendall’s tau test. Values of *P* < 0.05 were considered statistically significant. All statistical analyses were performed using the Statistica 12.0 PL software package.

## Results

At baseline, compared with groups B and C, patients belonging to group A showed higher values of HOMA1-IR, higher concentrations of uric acid, hsCRP, fibrinogen, homocysteine and lower concentrations of 25-hydroxyvitamin D. HsCRP levels were higher in group B than group C. The study groups did not differ from one another in terms of age, BMI, smoking status, SBP and DBP, plasma lipids and glucose (Table 1).

**Table 1:** Baseline characteristics of participants

Variable	Group A^a^	Group B^b^	Group C^c^	*P* values		
				A vs. B	A vs. C	B vs. C
Number (*n*)	19	19	21	–	–	–
Age [years; mean (SD)]	57 (10)	58 (6)	59 (10)	0.7108	0.5314	0.7073
Smokers (%)	32	26	29	0.7206	0.8358	0.8732
BMI [kg/m^2^; mean (SD)]	29.1 (4.6)	28.1 (4.0)	27.6 (3.8)	0.4792	0.2662	0.6875
SBP [mmHg; mean (SD)]	140 (11)	136 (12)	139 (12)	0.2913	0.7858	0.4347
DBP [mmHg; mean (SD)]	91 (8)	88 (6)	87 (8)	0.1993	0.1226	0.6600
Glucose [mg/dL; mean (SD)]	96 (10)	92 (9)	91 (8)	0.2032	0.0875	0.7119
HOMA1-IR [mean (SD)]	3.1 (0.8)	2.3 (0.8)	2.1 (0.6)	0.0039	0.0001	0.3738
Total cholesterol [mg/dL; mean (SD)]	265 (31)	269 (34)	273 (38)	0.7070	0.4730	0.7287
LDL-cholesterol [mg/dL; mean (SD)]	178 (28)	180 (25)	185 (30)	0.8177	0.4516	0.5726
HDL-cholesterol [mg/dL; mean (SD)]	49 (9)	52 (8)	50 (7)	0.2847	0.6956	0.4043
Triglycerides [mg/dL; mean (SD)]	170 (25)	159 (27)	164 (34)	0.2008	0.5324	0.6121
Testosterone [nmol/L; mean (SD)]	7.8 (1.3)	24.5 (10.2)	29.4 (8.7)	<0.0001	<0.0001	0.1094
Uric acid [mg/dL; mean (SD)]	5.1 (0.9)	4.1 (1.1)	4.0 (0.9)	0.0041	0.0004	0.7538
hsCRP [mg/L; mean (SD)]	4.0 (0.9)	3.2 (0.7)	2.6 (0.7)	0.0042	<0.0001	0.0101
Fibrinogen [mg/dL; mean (SD)]	403 (82)	329 (76)	310 (69)	0.0067	0.0004	0.4124
Homocysteine [μmol/L; mean (SD)]	32 (9)	23 (8)	22 (8)	0.0025	0.0006	0.6952
25-Hydroxyvitamin D [ng/mL; mean (SD)]	20 (7)	27 (8)	29 (7)	0.0068	0.0002	0.4043

HDL, high-density lipoprotein; HOMA1-IR, the homeostatic model assessment 1 of insulin resistance ratio; hsCRP, high-sensitivity C-reactive protein; LDL, low-density lipoprotein.

^a^Men with untreated low testosterone levels.

^b^Men with testosterone levels within the reference range receiving testosterone enanthane because of hypotestosteronemia.

^c^Drug-naive men with testosterone levels within the reference range.

No serious adverse effects of atorvastatin were reported and all participants completed the study. Daily caloric intake did not differ between the study groups [group A: 2250 (342) kcal; group B: 2,305 (370) kcal (*P* = 0.6371 vs. group A), group C: 2342 (320) kcal (*P* = 0.3850 vs. group A, *P* = 0.7364 vs. group B)]. There were no differences between the study groups in the average rate of energy expenditure during the activity [group A: 4.6 (0.8) metabolic equivalents; group B: 4.4 (0.7) metabolic equivalents (*P* = 0.3844 vs. group A), group C: 4.5 (0.6) metabolic equivalents (*P* = 0.6553 vs. group A, *P* = 0.6295 vs. group B)] as well as in the average level of effort [group A: 5.51 (0.28); group B: 5.53 (0.26) (*P* = 0.8208 vs. group A), group C: 5.59 (0.20) (*P* = 0.3015 vs. group A, *P* = 0.4159 vs. group B)].

In all study groups, atorvastatin reduced total and LDL-cholesterol. The drug did not affect HDL-cholesterol, triglycerides and plasma glucose. The impact on total and LDL-cholesterol was stronger, while post-treatment values were lower in groups B and C than in group A (Table 2).

**Table 2:** Plasma lipids, glucose homeostasis markers and the investigated cardiometabolic risk factors in atorvastatin-treated hypercholesterolemic men with either low or normal plasma testosterone levels

Variable	Group A^a^	Group B^b^	Group C^c^	*P* values		
				A vs. B	A vs. C	B vs. C
Glucose [mg/dL; mean (SD)]						
At the beginning of the study	96 (10)	92 (9)	91 (8)	0.2032	0.0875	0.7119
At the end of the study	98 (12)	94 (10)	92 (8)	0.2717	0.0682	0.4872
*P* value (post-treatment vs. baseline)	0.5802	0.5004	0.6876	–	–	–
HOMA1-IR [mean (SD)]						
At the beginning of the study	3.1 (0.8)	2.3 (0.8)	2.1 (0.6)	**0.0039**	**0.0001**	0.3738
At the end of the study	3.8 (0.7)^#^	2.2 (0.9)	2.0 (0.7)	**<0.0001**	**<0.0001**	0.3452
*P* value (post-treatment vs. baseline)	**0.0068**	0.7023	0.6219	–	–	–
Total cholesterol [mg/dL; mean (SD)]						
At the beginning of the study	265 (31)	269 (34)	273 (38)	0.7070	0.4730	0.7287
At the end of the study	227 (29)	185 (36)*	190 (30)*	**0.0003**	**0.0003**	0.6348
*P* value (post-treatment vs. baseline)	**0.0004**	**<0.0001**	**<0.0001**	–	–	–
LDL-cholesterol [mg/dL; mean (SD)]						
At the beginning of the study	178 (28)	180 (25)	185 (30)	0.8177	0.4516	0.5726
At the end of the study	140 (23)	99 (16)*	103 (17)*	**<0.0001**	**<0.0001**	0.4495
*P* value (post-treatment vs. baseline)	**0.0001**	**<0.0001**	**<0.0001**	–	–	–
HDL-cholesterol [mg/dL; mean (SD)]						
At the beginning of the study	49 (9)	52 (8)	50 (7)	0.2847	0.6956	0.4043
At the end of the study	53 (10)	54 (8)	53 (8)	0.7356	1.0000	0.6952
*P* value (post-treatment vs. baseline)	0.2032	0.4460	0.2034	–	–	–
Triglycerides [mg/dL; mean (SD)]						
At the beginning of the study	170 (25)	159 (27)	164 (34)	0.2008	0.5324	0.6121
At the end of the study	152 (30)	142 (32)	147 (29)	0.3270	0.5953	0.6071
*P* value (post-treatment vs. baseline)	0.0521	0.0852	0.0890	–	–	–
Testosterone [nmol/L; mean (SD)]						
At the beginning of the study	7.8 (1.3)	24.5 (10.2)	29.4 (8.7)	**<0.0001**	**<0.0001**	0.1094
At the end of the study	7.4 (1.2)	23.7 (9.8)	26.9 (9.1)	**<0.0001**	**<0.0001**	0.2910
*P* value (post-treatment vs. baseline)	0.3209	0.8067	0.3684	–	–	–
Uric acid [mg/dL; mean (SD)]						
At the beginning of the study	5.1 (0.9)	4.1 (1.1)	4.0 (0.9)	**0.0041**	**0.0004**	0.7538
At the end of the study	4.8 (1.1)	3.2 (0.9)*	3.4 (0.8)*	**<0.0001**	**<0.0001**	0.4641
*P* value (post-treatment vs. baseline)	0.3637	**0.0090**	**0.0278**	–	–	–
hsCRP [mg/L; mean (SD)]						
At the beginning of the study	4.0 (0.9)	3.2 (0.7)	2.6 (0.7)	**0.0042**	**<0.0001**	**0.0101**
At the end of the study	3.2 (0.8)	1.7 (0.6)*	1.4 (0.5)*	**<0.0001**	**<0.0001**	0.0929
*P* value (post-treatment vs. baseline)	**0.0064**	**<0.0001**	**<0.0001**	–	–	–
Fibrinogen [mg/dL; mean (SD)]						
At the beginning of the study	403 (82)	329 (76)	310 (69)	**0.0067**	**0.0004**	0.4124
At the end of the study	389 (74)	263 (87)*	251 (62)*	**<0.0001**	**<0.0001**	0.6157
*P* value (post-treatment vs. baseline)	0.5840	**0.0175**	**0.0058**	–	–	–
Homocysteine [μmol/L; mean (SD)]						
At the beginning of the study	32 (9)	23 (8)	22 (8)	**0.0025**	**0.0006**	0.6952
At the end of the study	31 (8)	16 (7)*	15 (6)*	**<0.0001**	**<0.0001**	0.6295
*P* value (post-treatment vs. baseline)	0.7195	**0.0068**	**0.0026**	–	–	–
25-hydroxyvitamin D [ng/mL; mean (SD)]						
At the beginning of the study	20 (7)	27 (8)	29 (7)	**0.0068**	**0.0002**	0.4043
At the end of the study	21 (8)	35 (10)*	37 (8)*	**<0.0001**	**<0.0001**	0.4872
*P* value (post-treatment vs. baseline)	0.6842	**0.0099**	**0.0014**	–	–	–

Values in bold are statistically significant.

HDL, high-density lipoprotein; HOMA1-IR, the homeostatic model assessment 1 of insulin resistance ratio; hsCRP, high-sensitivity C-reactive protein; LDL, low-density lipoprotein.

^a^Men with untreated low testosterone levels.

^b^Men with testosterone levels within the reference range receiving testosterone enanthane because of hypotestosteronemia.

^c^Drug-naive men with testosterone levels within the reference range.

*The impact of atorvastatin (percent changes from baseline after adjustment for baseline values) more pronounced than in group A.

^#^The impact of atorvastatin (percent changes from baseline after adjustment for baseline values) more pronounced than in groups B and C.

**Fig. 1:**
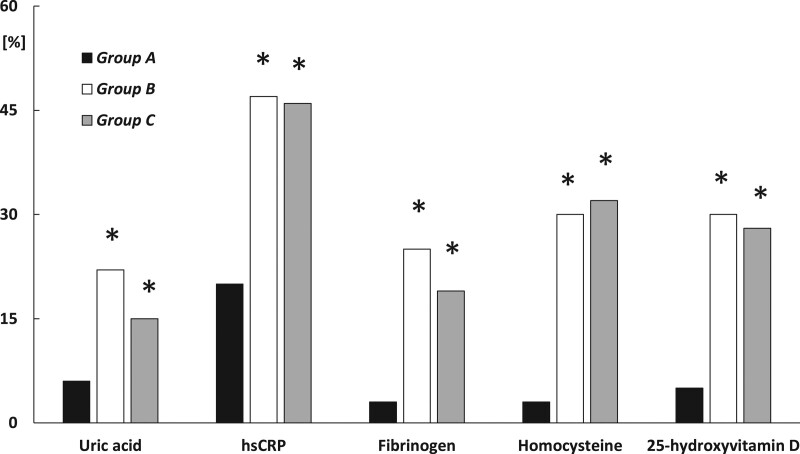
Percentage changes from baseline in cardiometabolic risk factors in atorvastatin-treated hypercholesterolemic patients with hypotestosteronemia or testosterone levels within the reference range. *P < 0.05 vs. group A.

In group A, atorvastatin use was associated with a decrease in hsCRP levels and with an increase in HOMA1-R. In patients with untreated low testosterone levels, uric acid, fibrinogen, homocysteine and 25-hydroxyvitamin D remained at a similar level throughout the study period. In groups B and C, uric acid, hsCRP, fibrinogen and homocysteine levels were lower after than before atorvastatin treatment. In both these groups, atorvastatin use was associated with an increase in 25-hydroxyvitamin D levels, while HOMA1-IR values did not differ between the first and last study day. The changes in uric acid, hsCRP, fibrinogen, homocysteine and 25-hydroxyvitamin D were more pronounced in groups B and C than in group A (Fig. 1), while the opposite relationship was found for HOMA1-IR. Groups B and C differed from group A in post-treatment values of HOMA1-IR, uric acid, hsCRP, fibrinogen, homocysteine and 25-hydroxyvitamin D (Table 2).

At the beginning of the study, concentrations of total and LDL-cholesterol correlated with plasma levels of uric acid [*r* values between 0.25 (*P* < 0.05) and 0.32 (*P* < 0.05)], hsCRP (*r* values between 0.29 (*P* < 0.05) and 0.43 (*P* < 0.001)], fibrinogen [*r* values between 0.26 (*P* < 0.05) and 0.41 (*P* < 0.001)], homocysteine (*r* values between 0.30 (*P* < 0.05) and 0.44 (*P* < 0.001)], and 25-hydroxyvitamin D [*r* values between −0.31 (*P* < 0.05) and −0.46 (*P* < 0.001)]. In group A, testosterone levels correlated with HOMA1-IR [*r* = −0.48 (*P* < 0.001)], and with plasma levels of uric acid [*r* = −0.34 (*P* < 0.05)], hsCRP [*r* = −0.39 (*P* < 0.001)], fibrinogen [*r* = −0.37 (*P* < 0.01)] and 25-hydroxyvitamin D [*r* = 0.28 (*P* < 0.05)]. The impact of atorvastatin on uric acid, hsCRP, fibrinogen, homocysteine and 25-hydroxyvitamin D did not correlate with baseline values or treatment-induced changes in plasma lipids and glucose homeostasis markers. In groups B and C, changes from baseline in uric acid, hsCRP, fibrinogen and homocysteine correlated positively [*r* values between 0.24 (*P* < 0.05) and 0.46 (*P* < 0.001)], while changes in 25-hydroxyvitamin D correlated negatively [group B: *r* = −0.35 (*P* < 0.01), group C: *r* = −0.38 (*P* < 0.01)] with their baseline levels. Moreover, the changes in uric acid, hsCRP, fibrinogen, homocysteine and 25-hydroxyvitamin D correlated with baseline testosterone levels [*r* values between 0.28 (*P* < 0.05) and 0.43 (*P* < 0.001)]. The remaining correlations were insignificant.

## Discussion

The major finding of the present study is that testosterone levels modulated the impact of atorvastatin treatment on plasma levels of cardiometabolic risk factors. The changes associated with atorvastatin use were much pronounced in patients with testosterone levels within the reference range than in subjects with low concentrations of this hormone. On the other hand, there were only small differences between patients with intact hypothalamic-pituitary-testicular axis activity and in those patients with an impaired testicular function who were effectively treated with exogenous testosterone preparations. The obtained results indicate that hormonal dysfunction itself, not the underlying disorder, is responsible for the worsening of statin action, as well as suggest that cardiometabolic effects associated with HMG-CoA reductase inhibitor therapy may be fully restored if testicular failure is compensated by testosterone supplementation. Between-group differences in the impact on cardiometabolic risk factors do not seem to be associated with daily caloric intake and physical activity, which were very similar in all treatment arms.

Despite the selection procedure aimed at creating study three arms similar to one another in terms of age, BMI, plasma lipid levels and blood pressure, untreated individuals with low testosterone levels differed from the remaining groups of patients in baseline circulating levels of uric acid, hsCRP, fibrinogen, homocysteine and 25-hydroxyvitamin D, as well as in the baseline degree of insulin sensitivity. Because these variables correlated with testosterone levels, their elevated levels seem to result from hypotestosteronemia. Taking into account high prediction accuracies of all these biomarkers [4,15–18], the obtained results suggest that impaired testosterone production makes men more susceptible to the development or progression of cardiometabolic disorders. Moreover, small differences in baseline values between both groups of subjects with testosterone levels within the reference range indicate that cardiometabolic risk may be minimized if subjects receive testosterone supplementation. Higher hsCRP levels in testosterone-treated men with testicular dysfunction than in untreated men with normal testosterone probably reflect limitations of intramuscular treatment with short-acting testosterone preparations in hormone replacement therapy. Certainly, we cannot totally exclude that the obtained results are partially related to the long-term effect of an unhealthy lifestyle or less stringent supervision by local healthcare providers of the patient’s non-pharmacological treatment (adherence to dietary recommendations and exercise) during three or more months preceding the beginning of the study.

Although triglyceride levels tended to decrease in all studied groups, the impact of atorvastatin did not reach the level of significance. This finding was probably a consequence of normal or only slightly elevated baseline concentrations of triglycerides in the investigated population because triglyceride-lowering effects of statins were found to depend on baseline levels of this lipid fraction [1,3]. The lack of correlations between cardiometabolic effects associated with atorvastatin use and its action on total and LDL-cholesterol levels, contrasted with the presence of such correlations at baseline. This suggests that although hypercholesterolemia increases the risk of cardiovascular and metabolic disorders, the reduction of this risk is associated with the improvement in both plasma lipids and with the independent inhibitory action of atorvastatin on the post-translational prenylation of small guanosine-5’-triphosphate-binding proteins, leukocyte function-associated antigen-1 intercellular adhesion molecule-1 interaction or nuclear factor-κB pathway [19,20]. Interestingly, all these pathways seem to be regulated by the hypothalamic-pituitary-testicular axis. Castration significantly increased the amounts of both RhoA and Rho-kinase in the rat cavernosal tissues and this effect was partially reversed by testosterone [21]. In men with type 2 diabetes, low testosterone levels enhanced leukocyte-endothelium cell interaction [22], while in cultured endothelial cells, testosterone downregulated nuclear factor-κB activity [23]. Similar effects on effector pathways of statins and testosterone and opposite effects of statins and testicular failure seem to explain why atorvastatin exerted a pluripotential beneficial effect on the investigated biomarkers in men with impaired hypothalamic-pituitary-testicular axis activity only if testicular dysfunction was compensated by the administration of the exogenous hormone. On the other hand, atorvastatin did not affect testicular function. Testosterone concentrations in the study groups were unaltered by statin therapy and this disparity with the results of previous studies [10,11] seems to be a consequence of using a moderate dose of atorvastatin in the present study. Unaltered testosterone levels were observed despite the fact that all participants were physically active and followed the exercise program during the whole period of the study. This finding is in line with previous observations that short bouts of maximal or near-maximal exercise lead to an increase in testosterone levels, while long-term, moderate-intensity, aerobic exercise either does not affect or even reduce testosterone levels (though it may increase dihydrotestosterone levels) [24,25].

Another interesting finding of the current study is that the unfavorable impact of untreated hypotestosteronemia on insulin sensitivity was aggravated by atorvastatin use, which contrasted with the neutral effect of this drug in the remaining study arms. It seems that in subjects without concomitant endocrine disorders, HMG-CoA reductase inhibitor-induced impairment in cellular glucose intake [26] is counterbalanced by anti-inflammatory properties of statins [27]. However, this balance may be disturbed by changes in testosterone production. In line with this hypothesis, impaired testicular function was found to induce low-grade systemic inflammation and to increase the production of tumor necrosis factor-α, interleukin-1β and interleukin-6, while exogenous testosterone preparations inhibited production of these cytokines [28,29]. Based on the obtained results, it may be assumed that subjects with low testosterone concentrations are at higher risk of statin-induced diabetes than other subjects but this risk does not differ from that observed in the general population if normal testosterone levels are restored.

Similar to low testosterone levels predicting increased all-cause and cardiovascular mortality, some retrospective studies and randomized trials suggest that testosterone replacement therapy may increase the risk of cardiovascular disease [30,31]. The lack of on-treatment testosterone levels in participants of these studies does not allow to exclude either inadequacy of treatment or supraphysiological testosterone levels. In our study, pleiotropic effects of atorvastatin in adequately supplemented hypogonadal men were similar to those observed in the control group. However, it remains to be established whether testosterone levels above the upper limit of normal modify cardiometabolic effects of statins or not.

Some other study limitations should be kept in mind during interpreting our findings. The most important one is the small sample size, being a consequence of difficulties in including in one research center a larger group of men meeting strict inclusion and exclusion criteria. We measured only surrogates of outcomes and therefore the obtained results should be supported in clinical trials assessing hard endpoints. Moreover, although the applied study protocol tried to limit the impact of random diurnal, seasonal and analytical variations in the hormones assayed, we cannot totally exclude the regression-toward-the-mean effect arising if a random variable is extreme on the first measurement but closer to the mean or average on further measurements. Furthermore, considering that all participants were treated with only one HMG-CoA reductase inhibitor, it remains uncertain whether our findings represent a ‘class effect’ or are a consequence of the unique properties of only one drug. Finally, it cannot be totally ruled out that the effect of atorvastatin, particularly in testosterone-naive men with low testosterone levels, might be stronger if atorvastatin was administered at higher doses than in the current study.

In conclusion, the impact of atorvastatin treatment on plasma lipids and on the measured cardiometabolic factors differed between the study groups, being much less pronounced in men with low testosterone levels than in their counterparts with testosterone levels within the reference range. Subjects with abnormally low production of this hormone benefited from atorvastatin use if they were concomitantly treated with exogenous testosterone preparations. Because of numerous study drawbacks, our research should be regarded as a pilot study and the obtained results should be verified in a large long-term clinical trial.

## Acknowledgements

The study was approved by the Bioethical Committee of the Medical University of Silesia.

### Conflicts of interest

There are no conflicts of interest.

## Footnotes

a Assuming a power of 80% and a significance level of 0.05, at least 17 men in each study arm had to be included to detect a 20% difference in all cardiometabolic risk factors assessed in the study.
